# SECTM1 coordinates immune microenvironments and multi-systemic disease pathophysiology

**DOI:** 10.3389/fimmu.2026.1819055

**Published:** 2026-05-18

**Authors:** Wenqing Yu, Yusheng Wu, Mingdong Ding, Li Guo, Ying Liu, Peiqing Yang, Jing Zhu, Daming Zhou, Yonglin Yang, Chengjing Gu

**Affiliations:** 1 Department of Infectious Diseases, The Affiliated Taizhou People’s Hospital of Nanjing Medical University, Taizhou School of Clinical Medicine, Nanjing Medical University, Taizhou, Jiangsu, China; 2 Department of Pharmacy, The Affiliated Taizhou People’s Hospital of Nanjing Medical University, Taizhou School of Clinical Medicine, Nanjing Medical University, Taizhou, Jiangsu, China

**Keywords:** biomarker, CD7/GITR axis, immune checkpoint, innate-adaptive crosstalk, secreted and transmembrane protein 1 (SECTM1), tumor microenvironment

## Abstract

Secreted and Transmembrane Protein 1 (SECTM1) is a versatile immunomodulatory protein that exists in both membrane-bound and soluble forms. Initially identified for its interaction with the T-cell receptor CD7, it was primarily regarded as a co-stimulatory molecule restricted to lymphoid signaling. Recent evidence has significantly expanded this paradigm, revealing that SECTM1 serves as a pivotal bridge between innate and adaptive immune responses. Beyond its classic role, SECTM1 interacts with alternative receptors like the glucocorticoid-induced TNFR-related protein to regulate macrophage activity and neutrophilic inflammation. This review highlights a major shift in our understanding of SECTM1: it acts as a multi-systemic regulator that influences the tumor microenvironment, metabolic homeostasis, and tissue regeneration. We systematically delineate the molecular mechanisms by which SECTM1 governs immune cell migration and activation across diverse pathologies, including cancer, cardiovascular disorders, and neurodegenerative diseases. By positioning SECTM1 as a marker of “immune-hot” tumors and a potential early diagnostic biomarker for systemic conditions, this work underscores its emerging clinical potential as a target for precision immunotherapy and regenerative medicine.

## 1 Introduction

Secreted and transmembrane protein 1 (SECTM1), also known as K12, is an immune-related transmembrane protein that was originally identified through its association with the T-cell surface molecule CD7 (1). The *SECTM1* gene is located upstream of *CD7* and encodes a protein that exists in both membrane-bound and secreted forms (2). Through its interaction with CD7, SECTM1 plays a pivotal role in T-cell co-stimulation, proliferation, and cytokine production, thereby functioning as an important molecular link between innate and adaptive immunity (3, 4). Accumulating evidence over the past decade has expanded this view, demonstrating that the biological activities of SECTM1 extend well beyond classical T-cell co-stimulation. Emerging studies increasingly implicate SECTM1 in shaping the tumor immune microenvironment, modulating host responses to pathogen infection, influencing metabolic and cardiovascular disorders, and contributing to tissue repair and regeneration.

In this review, we first delineate the molecular characteristics, expression regulation, and biological functions of SECTM1, with particular emphasis on its dual receptor interactions (CD7 and GITR) and isoform-specific features. We subsequently provide a systematic elaboration of its context-dependent roles across major disease categories, positioning SECTM1 as a pivotal “bridge molecule” linking immune responses with systemic pathophysiology. Within infectious diseases, SECTM1 functions as both a pro-inflammatory enhancer and a negative feedback regulator. In malignancies, its roles diverge into tumor-promoting or immune-beneficial functions depending on the unique tumor microenvironment. The reach of this molecule extends into cardiovascular and metabolic disorders, where emerging proteomic data associate SECTM1 with coronary heart disease and diabetic kidney disease. Furthermore, in tissue repair and neurodegenerative contexts, it demonstrates regenerative potential in corneal injury and serves as an early pre-diagnostic marker for amyotrophic lateral sclerosis. Finally, we critically evaluate current knowledge gaps, specifically the functional distinctions between soluble and membrane-bound isoforms, incomplete receptor signaling networks, and the lack of translational validation, and propose future research directions to address these deficiencies. By organizing the review within this framework, we aim to provide a comprehensive and accessible perspective on SECTM1 as a prototypical bridge molecule, underscoring its emerging clinical potential far beyond its traditional role in T-cell co-stimulation.

## 2 Molecular biology of SECTM1

### 2.1 Molecular characteristics and expression regulation

The human *SECTM1* gene is located on chromosome 17q25, spans approximately 14 kb, and encodes a 1.8-kb messenger RNA (2). This transcript gives rise to a 248-amino-acid protein whose hydrophobic profile is consistent with a type 1a membrane protein (2). Western blot analysis reveals that SECTM1 appears as a cluster of bands around 27 kDa, supporting its characterization as an integral membrane protein (2). In addition to its membrane-bound form, the N-terminal region can be proteolytically processed to generate an approximately 20-kDa soluble isoform that is secreted into the extracellular space (2). Immunofluorescence studies demonstrate that SECTM1 is predominantly localized in a perinuclear, Golgi-like compartment rather than being abundantly expressed on the cell surface (2). Expression profiling further shows that SECTM1 messenger RNA is most highly expressed in peripheral blood leukocytes and breast cancer cell lines (2). Consistently, immunohistochemical analyzes confirm strong protein expression in myeloid leukocytes, particularly granulocytes, whereas expression is minimal or absent in lymphocytes (2).

From an evolutionary perspective, the human genome contains a single *SECTM1* gene, whereas mice harbor two homologous genes, *Sectm1a* and *Sectm1b* (1, 5). These paralogs are located in close proximity on mouse chromosome 11 and encode proteins with substantial sequence similarity (5). Among them, SECTM1A exhibits the greatest homology and functional resemblance to human SECTM1 (5–7), suggesting that it may represent the principal murine counterpart for mechanistic studies. Intriguingly, a SECTM1 homolog has also been identified in the genome of a novel equine molluscum contagiosum-like virus, which encodes a SECTM1-like protein (8). This unexpected finding raises the possibility that SECTM1-related molecules may have been co-opted during evolution to modulate host–pathogen interactions.

The expression of *SECTM1* is tightly and multilayeredly regulated. Its promoter region contains binding sites for transcription factors including STAT1α/GAS, STAT3, and NF-κB, underscoring the complexity of its transcriptional control network (9). As a canonical interferon response gene, *SECTM1* is rapidly induced by multiple interferon subtypes. In human monocytic cells, IFN-γ robustly upregulates *SECTM1* expression through a STAT1-dependent pathway, and this induction occurs independently of *de novo* protein synthesis, a defining feature of early interferon response genes (3, 9).

Type I interferons (IFN-α/β) similarly stimulate *SECTM1* expression in human monocytic cells and mouse lung epithelial cells (7, 9). In contrast, lipopolysaccharide suppresses *SECTM1* expression in monocytes, highlighting stimulus-specific regulatory effects (9).

Epigenetic mechanisms further contribute to *SECTM1* regulation. In kidney renal papillary cell carcinoma, aberrant DNA methylation of the *SECTM1* locus is significantly associated with its expression level and with patient prognosis (10). In addition, 1,25-dihydroxyvitamin D3 has been reported to modulate *SECTM1* expression in placental trophoblast cells (11), indicating that hormonal signaling pathways may also influence its transcriptional dynamics.

### 2.2 Biological functions of SECTM1

The biological functions of SECTM1 are highly cell- and context-dependent, enabling it to exert diverse effects across distinct immune microenvironments (3, 12). One of its best-characterized receptors is CD7, which is expressed on the surface of T cells and NK cells and has been validated as a binding partner of SECTM1 (1). Through this interaction, the soluble form of human SECTM1 potently co-stimulates the proliferation and IFN-γ production of both CD4^+^ and CD8^+^ T cells (3). Notably, SECTM1-mediated signaling demonstrates synergistic activity: in the presence of CD28 co-stimulation, it markedly enhances IL-2 production following T-cell receptor activation, thereby amplifying adaptive immune responses (3).

Importantly, the functional repertoire of SECTM1 is not restricted to CD7-dependent signaling. Although both murine SECTM1A and SECTM1B can bind CD7, they exert opposing effects on T-cell activation, stimulatory versus inhibitory, and these divergent functions have been shown to occur independently of CD7 (5). Subsequent studies identified glucocorticoid-induced tumor necrosis factor receptor–related protein (GITR) as an alternative receptor for SECTM1A (5). Engagement of this receptor on macrophages activates the PI3K–Akt signaling pathway, leading to enhanced phagocytic and bactericidal activity and conferring protection in experimental models of sepsis (6). These findings highlight the receptor diversity and signaling plasticity underlying the context-specific immunoregulatory functions of SECTM1.

## 3 Role of SECTM1 in clinical diseases

### 3.1 Function in infectious diseases

SECTM1 exerts complex and context-dependent effects in infectious diseases, with its function shaped by pathogen type, tissue location, and the surrounding immune microenvironment. In murine models of lung infection, epithelial cell–derived SECTM1a, induced by type I interferons, displays dual regulatory properties. On the one hand, it binds activated neutrophils within infected lung tissue and promotes their production of the chemokine CXCL2, thereby establishing a positive feedback loop that amplifies neutrophilic inflammation (7). On the other hand, recent studies have demonstrated that SECTM1a can directly suppress IL-17A production by pulmonary γδ T cells. *In vivo* depletion of *Sectm1a* enhances γδ T-cell activity, increases neutrophil recruitment, and facilitates early bacterial clearance (13), suggesting that SECTM1a may function as a negative regulator to restrain excessive inflammatory responses. In systemic sepsis, SECTM1 plays a protective role by activating macrophages through GITR signaling. Reduced SECTM1 levels are associated with worse clinical outcomes (6), further underscoring its immunomodulatory capacity in severe infection.

In the setting of tuberculosis complicated by diabetes, SECTM1 has been identified as an immune-related biomarker through integrated bioinformatics and machine-learning approaches, together with cholesteryl ester transfer protein (CETP) and TYRO protein tyrosine kinase binding protein (TYROBP) (14). An early warning model constructed on the basis of these genes demonstrated favorable predictive performance (14). Clinically, *SECTM1* messenger RNA levels in patients with tuberculosis and diabetes were positively correlated with sputum acid-fast bacilli grading and declined during anti-tuberculosis treatment, supporting its potential utility as a marker of disease activity and therapeutic response (15).

In viral infection, secretome profiling of pediatric airway epithelial cells infected with human respiratory syncytial virus revealed specific induction of SECTM1, alongside emerging immunomodulatory mediators such as C-X-C motif chemokine ligand 6 (CXCL6), C-X-C motif chemokine ligand 16 (CXCL16), and colony-stimulating factor 3 (CSF3) (16). These findings further underscore the responsiveness of SECTM1 to pathogen-driven immune activation.

Under infectious conditions, the core bridging function of SECTM1 is defined by its ability to dynamically balance immune responses. It establishes an “inflammatory bridge” to facilitate neutrophil recruitment for pathogen clearance while simultaneously constructing a “buffer bridge” to suppress excessive immune activation and prevent collateral tissue damage. This adaptive capacity to modulate its functional output in response to microenvironmental cues underscores its pivotal role as a key molecular link between the immune system and clinical outcomes. As the focus shifts from exogenous pathogens to endogenous aberrant proliferation, the bridging role of SECTM1 within the tumor immune microenvironment reveals an even more distinct and complex landscape.

### 3.2 Role in malignancies

In the context of malignancy, the bridging function of SECTM1 is further expanded, primarily through its ability to mediate bidirectional communication between tumor cells and the immune microenvironment. As such, SECTM1 acts as a critical regulatory node that can shape immune responses toward either pro-tumorigenic or antitumor outcomes. SECTM1 acts as a double-edged regulator in cancer, serving as a critical intermediary between tumor cells and the immune microenvironment. It can directly influence tumor progression as well as shape antitumor immunity. In melanoma, tumor-derived SECTM1 recruits monocytes and macrophages through engagement of CD7 and activation of the PI3K pathway, thereby fostering a pro-tumorigenic microenvironment (17). In esophageal squamous cell carcinoma (ESCC), elevated SECTM1 expression promotes macrophage polarization toward an M2-like phenotype and drives tumor progression through C-C motif chemokine ligand 5 (CCL5)–dependent mechanisms, as demonstrated experimentally (18). In contrast, evidence from soft-tissue sarcoma suggests a potentially immunostimulatory role. Bioinformatic analyzes indicate that higher SECTM1 expression is associated with increased infiltration of activated CD8^+^ T cells and NK cells, implying enhanced antitumor immune activity; however, these findings remain to be validated experimentally (19). Consistently, pan-cancer analyzes have demonstrated that SECTM1 is enriched in “immune-hot” tumors across multiple cancer types (12). Both intratumoral and circulating SECTM1 levels have been associated with improved clinical outcomes and enhanced responsiveness to immune checkpoint inhibitor therapy, supporting its potential utility as a predictive biomarker in cancer immunotherapy (12).

Beyond its immunomodulatory effects within the tumor microenvironment, SECTM1 can directly regulate tumor cell behavior. In ESCC and glioblastoma, enforced SECTM1 expression enhances cancer cell proliferation, migration, invasion, and epithelial–mesenchymal transition (EMT)–like phenotypes (18, 20). In glioblastoma, these protumorigenic effects are primarily mediated through activation of the TGFβ1/Smad signaling pathway (20). Emerging evidence further suggests that SECTM1 may contribute to resistance to the multikinase inhibitor lenvatinib in hepatocellular carcinoma (21), highlighting its potential role in therapeutic refractoriness.

Clinically, SECTM1 expression has been associated with prognosis across multiple malignancies, including melanoma, kidney cancer, prostate cancer, and ESCC (10, 17, 18, 22). Importantly, its interaction with CD7 has opened new avenues for therapeutic innovation. For example, chimeric antigen receptor (CAR) T cells engineered using the extracellular domain of SECTM1 can selectively target CD7-positive malignancies while avoiding T-cell fratricide, providing a rational strategy for treating CD7-expressing hematologic cancers (23).

In summary, the bridging role of SECTM1 in malignancy presents a profound dual complexity: it can function as a “malignant bridge”, tethering tumor cells to immunosuppressive myeloid cells to drive disease progression, or alternatively, act as an “antitumor immune bridge” associated with the robust activation of cytotoxic lymphocytes. This functional specificity is governed by the tumor type and the distinct composition of the immune microenvironment, factors that profoundly influence both patient prognosis and the efficacy of immunotherapy. Notably, the regulatory reach of SECTM1 extends beyond the realms of cancer and infection; its bridging capacity also encompasses metabolic and cardiovascular disorders characterized by chronic, low-grade inflammation.

### 3.3 Cardiovascular and metabolic disorders

The universality of SECTM1 as a bridge molecule is strikingly evident in the context of cardiometabolic diseases. Although these conditions are distinct from classical infections or malignancies, their pathogenesis and progression are inextricably linked to chronic immune-mediated inflammation. In this setting, SECTM1 functions as a critical intermediary, tethering systemic immune status to localized target organ damage. Large-scale proteomic analyzes have identified SECTM1 as a potential biomarker and pathogenic mediator in new-onset onset coronary heart disease (CHD) (24). In the Jackson Heart Study, elevated plasma SECTM1 levels were significantly associated with an increased risk of incident CHD among Black participants (24). Moreover, the genetic variant rs116473040, which is linked to circulating SECTM1 levels, was correlated with the proportion of peripheral blood monocytes (24). Functional studies further demonstrated that recombinant SECTM1a increased the proportion of pro-atherogenic Ly6C^hi^ monocytes, suggesting a mechanistic pathway by which SECTM1 may contribute to atherosclerosis through modulation of monocyte abundance and subset distribution (24). In the context of metabolic disease, integrated bioinformatics analyzes combined with experimental validation have identified SECTM1 as a core candidate biomarker for the early detection of diabetic kidney disease and confirmed its upregulated expression in renal tissues (25). These findings extend the role of SECTM1 beyond classical immune regulation to cardiometabolic pathology.

Consequently, within the field of cardiometabolic research, the bridging role of SECTM1 underscores its function as a systemic immune modulator. By regulating mechanisms such as the distribution of circulating monocyte subsets, SECTM1 conveys fluctuations in peripheral immune status to specific target organs, including the vascular walls and kidneys. This recruitment and activation contribute directly to the pathogenesis of atherosclerosis and diabetic nephropathy, demonstrating how bridge molecules transcend traditional disease classifications by intrinsically linking systemic immune dysregulation with organ-specific pathologies. Furthermore, the physiological repertoire of SECTM1 extends beyond pathological regulation to encompass vital roles in tissue regeneration and the maintenance of homeostatic balance within the nervous system.

### 3.4 Tissue repair and neurodegenerative diseases

Beyond its immunological functions, SECTM1 has emerged as a regulator of tissue repair. In models of corneal injury, SECTM1 expression is markedly upregulated in the corneas of juvenile rhesus monkeys and promotes the migration and proliferation of limbal stem cells, a process that may be partially mediated by cell division cycle–associated protein 7 (26). In contrast, impaired corneal healing in aged rhesus monkeys is associated with insufficient SECTM1 expression. Local administration of recombinant SECTM1 significantly accelerates corneal re-epithelialization and improves corneal transparency in both aged animals and experimental mouse models (26). These findings highlight the therapeutic potential of SECTM1 in regenerative medicine.

In the context of neurodegeneration, large-scale prospective proteomic analyzes have identified SECTM1 as one of five significant prediagnostic biomarkers detectable approximately two decades before the clinical onset of amyotrophic lateral sclerosis (27). This early alteration suggests that SECTM1 may participate in long-standing immune or systemic processes that precede overt neurodegeneration, supporting its potential relevance in disease prediction and mechanistic investigation (27).

From driving corneal regeneration through specialized repair signaling to serving as a pre-diagnostic plasma biomarker for neurodegeneration, the involvement of SECTM1 in these diverse processes expands its conceptualization as a key player in cross-system pathology. These findings illustrate that SECTM1 does more than establish pathways between the immune system and disease; it acts as a universal molecular mediator, bridging expansive physiological and pathological landscapes, including development, regeneration, aging, and neuroimmunity.

## 4 Current limitations and critical knowledge gaps

**Figure 1:**
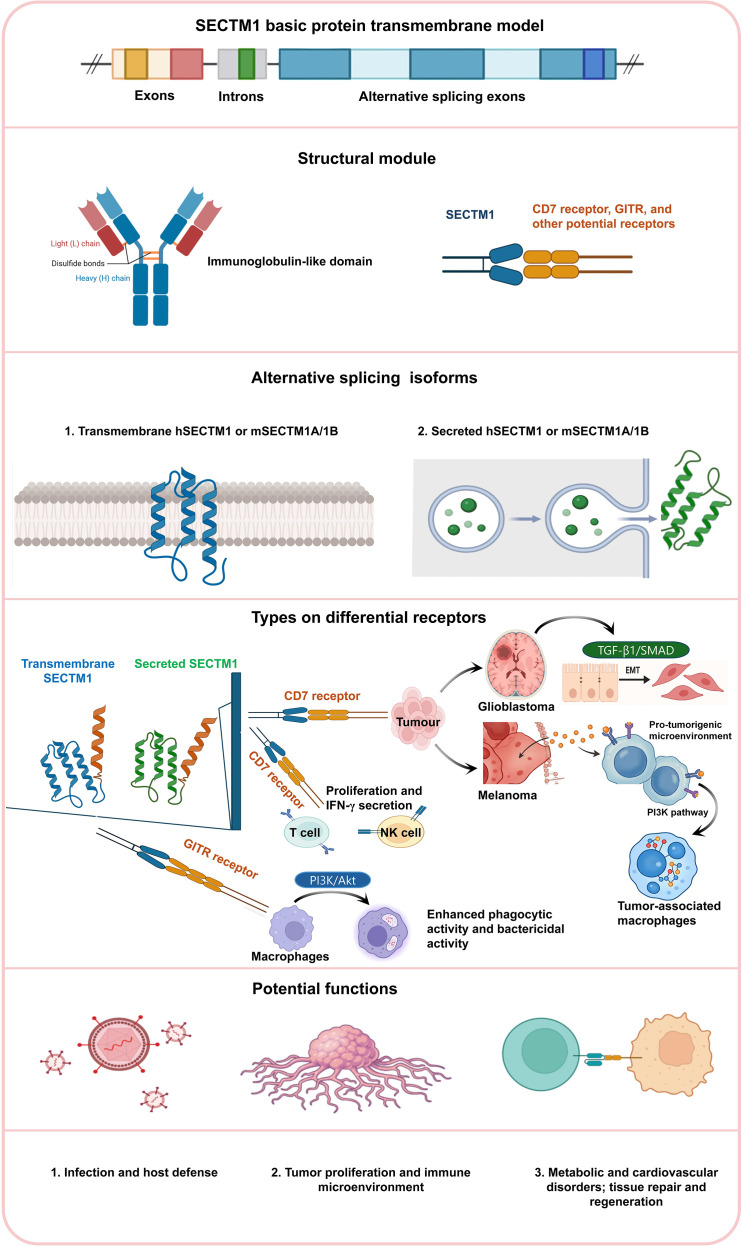
Overview of SECTM1 structure, isoforms, receptors, and biological functions. (1) Basic protein structure and splicing variants, including the transmembrane and secreted isoforms of human SECTM1 (hSECTM1) and murine SECTM1A/1B (mSECTM1A/1B), with annotations of exons, introns, and alternatively spliced regions. (2) Immunoglobulin-like domain, highlighting the light and heavy chains and disulfide bond architecture. (3) Receptors and signaling pathways, illustrating the interactions of SECTM1 with CD7, GITR, and other potential receptors, as well as downstream signaling cascades such as the PI3K–Akt pathway in macrophages and tumor-associated macrophages and the TGF-β1/Smad pathway in glioblastoma. (4) Cell type–specific outcomes, depicting the effects of SECTM1 on T cells, NK cells, macrophages, and tumor cells, including proliferation, IFN-γ secretion, enhanced phagocytic and bactericidal activity, epithelial–mesenchymal transition (EMT), and the establishment of a pro-tumorigenic microenvironment. (5) Overview of biological functions, summarizing the roles of SECTM1 in infection and host defense, tumor progression and immune microenvironment modulation, as well as in metabolic and cardiovascular disorders, tissue repair, and regeneration.

Despite substantial progress in elucidating the biological functions of SECTM1, important gaps remain in our understanding of its molecular heterogeneity, receptor signaling complexity, and context-dependent roles across diverse diseases (**Table 1**, **Figure 1**).

**Table 1:** Functional potential of SECTM1.

Functional category		Major roles summary	References
Immunoregulatory Functions	Modulation of T Cell Functions	Functions as a ligand for CD7, costimulating T cell proliferation and IFN-γ production in synergy with CD28. Distinct isoforms (SECTM1A/B) exert co-stimulatory or inhibitory effects on T cell activation.	(3, 5)
	Modulation of Macrophage Functions	Binds to and activates Glucocorticoid-Induced TNFR-Related Protein (GITR), thereby augmenting the phagocytic and bactericidal capabilities of macrophages, which exerts a protective effect in sepsis.	(6)
	Modulation of Neutrophil Functions	In *pneumococcal pneumonia* caused by *Streptococcus* pneumoniae, airway epithelial cell-derived Sectm1a binds to activated neutrophils and stimulates CXCL2 production, thereby potentiating neutrophilic inflammation.	(7)
		Conversely, the depletion of Sectm1a facilitates neutrophil recruitment and augments bacterial clearance in a *pneumococcal pneumonia* model through the modulation of γδ T cell functions.	(13)
	Chemoattraction of Monocytes/Macrophages	Tumor cell-derived SECTM1 interacts with CD7 on monocytes and activates the PI3K signaling pathway, facilitating monocyte migration and modulating the tumor microenvironment.	(17)
	Interferon Early Response Gene	In human monocytes, the expression of SECTM1 mRNA is rapidly upregulated by Type I and Type II interferons, while it is downregulated by lipopolysaccharide (LPS).	(9)
	Viral Immune Response	Within the airway epithelium infected by human respiratory syncytial virus (hRSV), SECTM1 is secreted as a novel immunomodulatory protein.	(16)
Tissue Repair	Promotion of Corneal Healing	In non - human primates, SECTM1 plays a critical role in corneal epithelial wound healing, promoting the migration and proliferation of limbal stem cells. The topical administration of SECTM1 enhances wound closure and transparency in aged corneas.	(26)
Disease Association & Biomarker Potential	Cancer Progression and Prognosis	In esophageal squamous cell carcinoma (ESCC), a high level of SECTM1 expression facilitates cancer cell proliferation, migration, and invasion, as well as M2 macrophage polarization, and is correlated with a poor prognosis.	(18)
		In glioblastoma multiforme (GBM), SECTM1 facilitates tumor progression and epithelial - mesenchymal transition (EMT) - like processes through the TGFβ1/Smad signaling pathway.	(20)
		A potential prognostic biomarker for kidney renal papillary cell carcinoma (KIRP)	(10)
	Predictive Biomarker for Immunotherapy	Highly expressed in "immuno-hot" tumors and exhibits a correlation with a more favorable response to immune checkpoint inhibitors across multiple cancer types.	(12)
	Gene Associated with Drug Resistance in Liver Cancer	The expression of it is upregulated in hepatocellular carcinoma (HCC) cells following lenvatinib treatment, indicating a possible correlation with drug resistance.	(21)
	Diagnostic and Risk Prediction Biomarker	A prospective diagnostic biomarker for diabetic kidney disease (DKD)	(25)
		A prospective diagnostic biomarker for the comorbidity of diabetes mellitus and tuberculosis (DM-TB)	(14)
		In patients with tuberculosis-diabetes mellitus (TB-DM), the mRNA level exhibits a positive correlation with the grade of sputum acid-fast bacilli.	(15)
		A component of an immune - related prognostic gene signature in soft tissue sarcoma (STS)	(19)
		Identified as a candidate susceptibility gene within blood exosomes associated with the risk of prostate cancer	(22)
	Viral-Encoded Homolog	An equine molluscum contagiosum-like virus (EMCLV) encodes a mammalian SECTM1 homolog, which potentially disrupts host immune signaling.	(8)
Therapeutic Exploration	Targeting Domain for CAR-T Therapy	The extracellular domain of SECTM1 was employed to engineer CD7-targeting CAR-T cells. These cells selectively eradicate CD7-high malignant cells, thereby facilitating the enrichment and proliferation of CD7-low/negative CAR-T subsets.	(23)

### 4.1 Functional distinction between soluble and membrane-bound isoforms

Accumulating evidence supports the existence of two distinct forms of SECTM1: a membrane-bound type I transmembrane protein and a soluble isoform generated through proteolytic cleavage (2). However, most studies have focused on their overall activity, with limited efforts to systematically compare their isoform-specific functions. The soluble form, by virtue of its diffusible nature, may regulate immune responses at a distance by establishing chemotactic gradients or mediating paracrine signaling. In contrast, the membrane-bound isoform is more likely to mediate direct juxtacrine signaling at sites of cell–cell contact, such as immune synapses or tumor–stromal interfaces. A detailed delineation of the distinct signaling pathways, receptor interactions, and context-specific functions of these isoforms is urgently needed. Such insights are not only of mechanistic importance but are also essential for the rational design of precision therapies that selectively target SECTM1 or its downstream pathways while preserving its beneficial physiological functions.

### 4.2 Receptor selectivity and signaling plasticity

Although CD7 is a well-established receptor for SECTM1 and GITR has been identified as an alternative receptor in murine systems, the full spectrum of SECTM1-interacting partners remains incompletely characterized (1, 5). Several key questions remain unresolved. These include whether GITR functions as a bona fide receptor for SECTM1 in humans; how the relative binding affinities of SECTM1 for CD7 and other candidate receptors vary across physiological and pathological contexts; which downstream signaling pathways are activated upon receptor engagement (e.g., PI3K–Akt, TGF-β1/Smad, or potentially JAK/STAT pathways); and how cell type–specific receptor expression patterns, such as CD7 on T and NK cells versus GITR on macrophages, shape functional outcomes. Notably, the observation that murine SECTM1A and SECTM1B exert opposing effects on T-cell activation independently of CD7 strongly suggests the existence of additional, as yet unidentified receptors or co-receptors (5). Elucidating this receptor network and its context-dependent signaling dynamics will be essential for a comprehensive understanding of SECTM1 biology and for the development of targeted therapeutic strategies.

### 4.3 Mechanistic basis of functional duality in cancer

A particularly intriguing yet incompletely understood feature of SECTM1 biology is its pronounced functional duality in malignancies. While SECTM1 exhibits tumor-promoting activity in certain cancers (e.g., melanoma, ESCC, and glioblastoma) (17, 18, 20), it is also associated with “immune-hot” phenotypes and favorable responses to immunotherapy in others (e.g., soft-tissue sarcoma and pan-cancer analyzes) (12, 19). To reconcile these seemingly contradictory observations, several interconnected, yet still underexplored, mechanisms should be considered. First, the cellular source of SECTM1 appears to be critical. Tumor cell–derived SECTM1, as reported in melanoma and ESCC, primarily targets immune cells, particularly monocytes and macrophages, via CD7 engagement, thereby promoting M2-like polarization and immunosuppressive microenvironments (17, 18). In contrast, SECTM1 produced by activated immune or stromal cells within inflamed tumor niches may function as a co-stimulatory or chemotactic signal that enhances lymphocyte recruitment and antitumor immunity. Second, receptor availability and cellular context are likely to shape downstream effects. Variability in CD7 expression across immune cell subsets, together with the potential involvement of alternative receptors such as GITR, may determine whether SECTM1 signaling leads to immune activation, suppression, or cell recruitment (23). Third, SECTM1 may exert direct tumor-intrinsic effects. In glioblastoma, for example, SECTM1 activates the TGF-β1/Smad pathway to promote epithelial–mesenchymal transition and invasive behavior (20). In such contexts, these direct pro-tumorigenic effects may predominate over its immunomodulatory functions. Finally, isoform-specific expression may further contribute to this functional heterogeneity. Although direct evidence remains limited, differential expression of soluble versus membrane-bound SECTM1 across tumor types or disease stages may influence receptor engagement and downstream signaling dynamics (17). These observations highlight the need for systematic investigation using cell type–specific models, single-cell multi-omics approaches, and functional assays capable of resolving isoform- and receptor-specific contributions. Such efforts will be essential to delineate the context-dependent roles of SECTM1 and to inform the rational development of targeted therapeutic strategies.

## 5 Translational challenges and future directions

The translation of SECTM1 research into clinically viable diagnostics and therapeutics faces several substantial challenges. First, SECTM1 exists in both soluble and membrane-bound forms with potentially distinct and even opposing functions. This heterogeneity necessitates the development of therapeutic strategies, such as neutralizing antibodies or small molecules, with high isoform specificity to selectively inhibit pathogenic signaling while preserving physiological functions. Second, leveraging its immunoregulatory properties, chimeric antigen receptor T-cell (CAR-T) therapies based on SECTM1 have emerged as a promising approach, particularly for CD7-positive hematological malignancies (23). These strategies typically utilize the extracellular domain of SECTM1 as the antigen-recognition module, enabling targeted elimination of CD7-expressing tumor cells (23). An additional advantage is the potential to enrich CAR-T products for CD7-negative or low-expressing T-cell subsets, thereby mitigating fratricide and improving cell persistence (23). However, several critical limitations must be considered. Because SECTM1 and CD7 are broadly expressed on normal immune cells, including T and NK cells, targeting this axis carries a significant risk of “on-target, off-tumor” toxicity, which may result in severe immunosuppression or long-term immune deficiency (2, 23). Moreover, current preclinical models have inherent limitations: mice lack a direct homolog of human SECTM1, and the functions of murine Sectm1a cannot be readily extrapolated to humans. The development of more clinically relevant systems, such as humanized SECTM1 transgenic models or patient-derived organoid–immune co-culture platforms, is therefore urgently needed to improve translational reliability. In addition, although SECTM1-based CAR-T approaches have demonstrated encouraging antitumor activity and reduced fratricide in preclinical settings, their long-term safety, susceptibility to antigen escape, and manufacturing complexity require rigorous evaluation. Complementary strategies, including antibodies targeting the SECTM1–CD7 interaction or small molecules modulating downstream signaling pathways, also hold therapeutic promise. Nevertheless, these approaches face notable challenges, particularly given that SECTM1 lacks intrinsic enzymatic activity, complicating conventional drug design. Overall, advancing SECTM1 toward clinical application will require a balanced and innovative approach that prioritizes safety, specificity, and mechanistic precision, while systematically addressing existing translational barriers.

## 6 Summary

SECTM1 is a distinctive immune-related transmembrane protein that occupies a central position in the regulation of immune cell activation, migration, and functional polarization through interactions with receptors such as CD7 and GITR. Beyond shaping the immune microenvironment, SECTM1 can directly promote malignant phenotypes in certain tumor types, while in other contexts it is enriched in immune-inflamed tumors and associated with improved responsiveness to immunotherapy. These dual and context-dependent effects underscore its complex role at the interface of tumor biology and immune regulation. In addition to cancer, SECTM1 has been implicated in infectious diseases, CHD, metabolic disorders, and tissue repair, highlighting its broad relevance across multiple physiological and pathological processes. Nevertheless, the precise mechanisms underlying its diverse functions remain incompletely understood. Future efforts should prioritize the integration of mechanistic studies with translational and clinical research, leveraging advanced omics technologies and refined animal models to delineate its regulatory networks. The development of diagnostic and therapeutic strategies targeting SECTM1 may ultimately enable its application in precision immunotherapy, infection management, and regenerative medicine.

Taken together, SECTM1 represents a key molecular link between immune regulation and multisystem disease. Continued investigation into its biological roles and clinical implications is likely to advance both mechanistic insight and therapeutic innovation.
